# Bridging the Gap Between Public Health and Political Science to Study the Populist Radical Right in its Multiple Manifestations: A Response to Recent Commentaries

**DOI:** 10.34172/ijhpm.2021.65

**Published:** 2021-06-26

**Authors:** Chiara Rinaldi, Marleen PM Bekker

**Affiliations:** ^1^Department of Health Services Research and Policy, London School of Hygiene and Tropical Medicine, London, UK.; ^2^Health and Society Group, Wageningen University & Research, Wageningen, The Netherlands.

 Since the publication of our paper *“A Scoping Review of Populist Radical Right Parties’ Influence on Welfare Policy and its Implications for Population Health in Europe,”* the relationship between the populist radical right (PRR) and population health has been more apparent than ever. PRR parties and leaders responded to the coronavirus disease 2019 (COVID-19) pandemic with various expressions of mistrust of science and medical experts, denialism, and stigmatization of ‘outsiders.’^1,2^ The pandemic also gave rise to new coronavirus-sceptic movements in wider society that connected to existing anti-vaccination and far right groups. In some countries this has led to the politicisation of the pandemic and the evidence-based measures to protect individuals and communities from infection, including physical distancing measures and the wearing of face coverings, with serious effects on incidence rates and population health.

 This critical juncture deepens the urgency of a research agenda that transcends the boundaries between public health and political science, deconstructing how ideas, interests and institutions affect health and vice versa. Using the excellent contributions in the 11 commentaries to our scoping review, we reconstruct the different meanings and manifestations of the PRR as described in the original paper. Although we acknowledge that there are limitations to our research design, such as the use of welfare policies as a proxy for population health,^3^ we will use this correspondence as an opportunity to go beyond this particular study to suggest a number of avenues for future research.

 We distinguish between three main common themes emerging from the commentaries. Gradually zooming out from the original paper, we first discuss the definitions and scope of populism and welfare chauvinism.^1,4-6^ We secondly reflect on the historic role of mainstream parties in welfare retrenchment, and how this relates to PRR discourses of the ‘(un)deserving.’^4,5,7^ Thirdly, we discuss the relevance of the PRR in relation to other policy areas that affect population health and health equity.^8-10^

## Global Manifestations, Definitions and Scope of Populism and Welfare Chauvinism

 Our scoping review revealed strong indications of welfare chauvinism, emphasising the nativist elements of the PRR agenda. Perhaps the most prominent contribution of the commentaries was a focus on populism – the hypothetical division between the ‘people’ and a corrupt elite.^1,4,11^ While useful, De Cleen and Speed emphasise that the term *populism* – which can be attributed to parties of either sides of the political spectrum – should not be conflated with the ideological core of PRR parties, which is mostly based on nativism.^5^ However, as Felder et al note, by widening the scope of research we can study alternative manifestations of populism as broader movements in society that are not directly captured when studying PRR political parties, but also influence public opinion and decision-making about health and welfare.^4^ Clark and Patterson point out that Latin American populist leaders, mostly within socialist parties, do not emphasise nativist welfare policies but rather use more inclusive anti-imperialist politics.^11^ There also appear to be differences between the policy positions and strategies of smaller PRR parties in Western Europe compared to dominant single-party PRR governments in Central and Eastern Europe.^6^ Making distinctions between different manifestations of populism is crucial for getting a better understanding of both the direct and indirect impacts of PRR parties and rhetoric.

 The concept of welfare chauvinism has also evolved towards a more refined typology that does more justice to alternative manifestations outside (Western) Europe that were not investigated in our scoping review sample. PRR parties’ appeals to welfare chauvinism are more nuanced and can be distinguished in three types.^6,12^ While many Western European PRR parties advocate for increased welfare spending for the native ‘in-group’ while excluding a fabricated ‘out-group’ (mostly immigrant groups and ethnic minorities, but in some countries this is also extended to members of the LGBTQ+ community), there are other parties and leaders that prefer ‘liberal chauvinism.’ *Liberal chauvinism* is a position that favours welfare retrenchment for all, but particularly affecting the ‘out-group’ that is thus more vulnerable to any negative effects. A third position popular among Eastern European PRR leaders is *welfare populism*, which aims to increase access to the welfare state for the common ‘people,’ while rejecting parts of it that are considered to serve the ‘elite.’^6^ These three positions on the welfare state that are adopted by different PPR parties can be expected to have differential impacts on population health and health inequities that require further empirical analysis.

## Welfare Retrenchment Policies and Reframing the “Undeserving”

 A second issue we draw from the commentaries is a critical reflection on the actual influence of PRR on welfare retrenchment. In multi-party systems, mainstream (usually conservative) coalition parties tend to have more influence than emerging PRR parties and movements. Especially in light of the history of neo-liberal welfare state reform in Western countries long before PRR parties became demarginalised, the policy influence of PRR parties in office does in itself not explain health system and welfare retrenchment.^4,7^ Interestingly, the most prominent exponents of the PRR ideology are in countries where ‘PRR practitioners’ operate *within* mainstream conservative political parties, including Donald Trump in the United States and Jair Bolsonaro in Brazil. By re-iterating and reframing common PRR discourses of the ‘deserving’ and ‘undeserving,’ mainstream parties may not only accommodate PRR positions to captivate some of the PRR electorate, but also to legitimate further retrenchment policies. The accommodation of PRR discourses by mainstream parties might in fact be an inverse accommodation of ongoing welfare retrenchment. In this context, the indirect and invisible impacts of PRR presence may be of greater influence on health, and particularly health equity, as Clavier et al rightly point out.^8^

## PRR Influence on Population Health Through Economic and Financial Policies

 While theoretically welfare policies seem more proximal to health and thus less subject to complex causal chains and uncertainties than distal determinants, empirical observations, re-affirmed by the COVID-19 crisis, indicate a relation between trade, labour market and fiscal policies and health. In this light, PRR policy preferences in these areas could be particularly harmful for already disadvantaged population groups, such workers on flexible contracts, small business owners and temporary migrant workers.^7-10^ The European Union (EU) represents contradictory incentives. On the one hand potential protective power from EU judicial institutions might prevent PRR policies from being implemented, while on the other hand the single market encourages labour migration under conditions of limited social rights for migrant workers in countries of destination, exposing them disproportionally to risks (eg, COVID-19) without proper care (eg, equal access to vaccines).

 In line with Labonté and Baum, we agree that the PRR’s excessive focus on economic (and border) protectionism and the rejection of international agreements (ranging from trade agreements to agreements to protect public health, human rights and environmental sustainability) should be included in further analyses of the PRR’s impacts on health and health equity.^8,9^ In addition to this, future analysis should also include the exclusionary identity politics of PRR parties and denial of public health and medical expertise.^1,2,9,11^ We welcome Stronks and Agyemang recommendation to apply an interdisciplinary systems approach that maps both downstream and upstream determinants of PRR influence on health, and the ways in which they are interrelated.^11^

## Some Implications for Future Research

 As evidenced by the rich debate that was sparked by our initial contribution, PRR parties and their policies are an important and timely public health research topic. The COVID-19 pandemic served as a real-world example of how PRR parties and leaders handle a major (health) crisis. The lessons we can draw from the commentaries point towards a general populist trend across many different countries and continents, yet with many different faces and policy implications, ranging from negligible in the margins to absolute majorities causing constitutional impact. Multiple research agendas emerge from the commentaries, either zooming in or zooming out from PRR parties as object of study. Zooming in requires follow up studies comparing PRR parties’ inner characteristics, positions and policies and their structural impacts, considering institutional enablers or disablers such as veto points in constitutional and health systems. Global analyses of PRR parties will allow us to better understand how and why the PRR varies across countries, and most importantly what the consequences are in different settings and for different populations. The recent book *“The Populist Radical Right and Health: National Policies and Global Trends”* edited by Michelle Falkenbach and Scott Greer is the first to do this in-depth.^12^ Zooming out from the PRR political party as object of study, future studies could incorporate broader manifestations of populism in society, such as vaccine scepticism and climate change denialism, and their possible impacts on health and equity.

 For the development of a comprehensive research agenda that addresses the complexity of political landscapes, party politics and policy-making processes, interdisciplinary collaborations are necessary. We therefore join previous calls for the consolidation of a ‘public health political science’ that benefits from theory and methods from both disciplines. Different fora need to be brought together in order to advance this subfield of research. The integration of public health with political science is for example already on the agenda of the European Public Health Association, mainly through the Public Health Policy and Politics section, and the American Political Science Association Health Politics and Policy section. We would also like to thank the editorial board of the International Journal of Health Policy and Management for facilitating this meaningful debate, and all authors who have contributed. We highly encourage and promote interdisciplinary empirical and normative research investigating any political determinant of health grounded in a firm, but not dogmatic, moral basis and substantiated by robust and real-world evidence.

## Ethical issues

 Not applicable.

## Competing interests

 Authors declare that they have no competing interests.

## Authors’ contributions

 CR prepared to first draft which was revised by MPB. Both authors equally contributed to subsequent drafts.

## References

[1] McKee M, Gugushvili A, Koltai J, Stuckler D (2021). Are populist leaders creating the conditions for the spread of COVID-19? Comment on “a scoping review of populist radical right parties’ influence on welfare policy and its implications for population health in Europe. ” Int J Health Policy Manag.

[2] Falkenbach M, Greer SL (2021). Denial and distraction: how the populist radical right responds to COVID- 19; Comment on “a scoping review of populist radical right parties’ influence on welfare policy and its implications for population health in Europe. ” Int J Health Policy Manag.

[3] Muis J (2021). Who deserves welfare and who does not? Comment on “a scoping review of populist radical right parties’ influence on welfare policy and its implications for population health in Europe. ” Int J Health Policy Manag.

[4] Felder M, Wallenburg I, Kuijper S, Bal R (2021). Taking the relationship between populism and healthcare seriously: a call for empirical analysis rather than moral condemnation comment on “a scoping review of populist radical right parties’ influence on welfare policy and its implications for population health in Europe. ” Int J Health Policy Manag.

[5] De Cleen B, Speed E (2021). Getting the problem definition right: the radical right, populism, nativism and public health: Comment on “a scoping review of populist radical right parties’ influence on welfare policy and its implications for population health in Europe. ” Int J Health Policy Manag.

[6] Moise AD (2021). Researching the welfare impact of populist radical right parties comment on “a scoping review of populist radical right parties’ influence on welfare policy and its implications for population health in Europe. ” Int J Health Policy Manag.

[7] Bambra C, Lynch J (2021). Welfare chauvinism, populist radical right parties and health inequalities: Comment on “a scoping review of populist radical right parties’ influence on welfare policy and its implications for population health in Europe. ” Int J Health Policy Manag.

[8] Clavier C, Martin E, Gagnon F (2021). Issue competition and the social construction of target populations: alternative suggestions for the study of the influence of populist radical right parties on health policy and health outcomes: cComment on “a scoping review of populist radical right parties’ influence on welfare policy and its implications for population health in Europe. ” Int J Health Policy Manag.

[9] Labonté R, Baum F (2021). Right wing politics and public policy: the need for a broad frame and further research: Comment on “a scoping review of populist radical right parties’ influence on welfare policy and its implications for population health in Europe. ” Int J Health Policy Manag.

[10] Stronks K, Agyemang C (2021). The multifaceted pathways linking populism to ethnic minority health: Comment on “a scoping review of populist radical right parties’ influence on welfare policy and its implications for population health in Europe. ” Int J Health Policy Manag.

[11] Clark MA, Patterson A (2021). Populism and health policy in Latin America: Comment on “a scoping review of populist radical right parties’ influence on welfare policy and its implications for population health in Europe. ” Int J Health Policy Manag.

[12] Falkenbach M, Greer SL. The Populist Radical Right and Health: National Policies and Global Trends. New York: Springer; 2021.

